# Concentration effects in the base-catalyzed hydrolysis of oligo(ethylene glycol)- and amine-containing methacrylic monomers

**DOI:** 10.1080/15685551.2016.1231034

**Published:** 2016-09-14

**Authors:** Oleg A. Kazantsev, Dmitry V. Orekhov, Alexey P. Sivokhin, Denis M. Kamorin, Maria V. Savinova

**Affiliations:** ^a^ Department of Chemical Engineering, Nizhny Novgorod State Technical University n.a. R.E. Alekseev, Nizhny Novgorod, Russia; ^b^ Department of Macromolecular Compounds and Colloid Chemistry, Lobachevsky State University of Nizhny Novgorod, Nizhny Novgorod, Russia

**Keywords:** Oligo(ethylene glycol) methacrylates, 3-(N,N-dimethylaminoethyl) methacrylate, 2-hydroxyethyl methacrylate, base-catalyzed hydrolysis, kinetics, structure of a solution

## Abstract

Concentration effects in the base-catalyzed hydrolysis of water-soluble methacrylates (3-(N,N-dimethylaminoethyl) methacrylate (DMAEMA), 2-hydroxyethyl methacrylate (HEMA) and oligo(ethylene glycol) methacrylates (OEGMAs)) have been studied. These monomers are rapidly hydrolyzed in the presence of bases at the room temperature in dilute aqueous solutions, but the reaction rate decreases sharply in highly concentrated solutions. A clear correlation was found between a form of the viscosity isotherm for DMAEMA solutions and the concentration dependence of the autocatalytic hydrolysis rate which indicates the connection of process kinetics with the structure of solutions. These data should be considered when carrying out homo- and copolymerization of the previously mentioned monomers in aqueous solutions.

## 1. Introduction

3-(N,N-Dimethylaminoethyl) methacrylate (DMAEMA), 2-hydroxyethyl methacrylate (HEMA) and oligo(ethylene glycol) methacrylates (OEGMAs) are the most commonly used water-soluble methacrylic esters. Water-soluble copolymers of OEGMAs (with the different ethoxylation degree, having terminal methoxy or hydroxy groups) are used as superplasticizers for concrete compositions, as superabsorbents, have a good potential in pharmaceutics.[1,2] HEMA copolymers are used for producing coatings and adhesives,[3,4] DMAEMA copolymers are effective flocculants and additives to paper.[5,6] In most cases, the preparation of such polymers is carried out in aqueous media. Furthermore, the monomers such as OEGMAs or DMAEMA salts are often obtained, stored and used in the form of concentrated aqueous solutions.[7]

It is known that esters in the presence of water have different resistance towards hydrolysis depending on the structure and conditions.[8,9] Bases are effective catalysts for the hydrolysis of esters.[8,10] Bases may be used to adjust the pH of aqueous solutions containing water-soluble methacrylic esters. Amine-containing methacrylates contain basic groups capable of self-catalyzing hydrolysis. Ethoxylated methacrylates, in some cases, are copolymerized in water with amine-contaning methacrylic monomers (DMAEMA or N-[3-(dimethylamino)propyl]methacrylamide),[11,12] i.e., when the system contains free amino groups. In this connection, the study of hydrolytic stability of the water-soluble amine-containing and ethoxylated (meth)acrylates in the presence of basic catalysts is very topical.

The mechanism and kinetics of the base-catalyzed hydrolysis of esters in dilute solutions are studied in detail.[13,14] According to the classical kinetic equation[15] the rate of the base-catalyzed hydrolysis of esters is proportional to the concentration of an ester and hydroxyl ions.

$$R_{h}=k\left[Ester\right]\left[OH^{-}\right]$$

The base-catalyzed hydrolysis of the alkyl (meth)acrylates was studied.[16–19] Because of the low solubility of the alkyl (meth)acrylates in water the reaction was studied in binary water-organic solvents or at very low concentrations in water. Previously, it was shown that hydroxyalkyl (meth)acrylates and alkoxyethyl methacrylates in neutral dilute aqueous solutions in the presence of buffer additives at 20 °C are not appreciably hydrolyzed and in a slightly alkaline medium (pH 8.8) half-life is 12 days (for ethylene glycol acrylate), 40 days (for propylene glycol acrylate), 33 days (2-ethoxyethyl methacrylate) [19]; the hydrolysis of DMAEMA in alkaline medium [20,21] can proceed rapidly, while the rate of the autocatalytic hydrolysis of DMAEMA markedly decreases when its concentration in aqueous solutions increases.[22] The hydrolysis of OEGMAs has not previously been studied.

The purpose of this work was to reveal and compare features of concentration effects in the base-catalyzed hydrolysis of the industrial water-soluble methacrylic esters: amine-containing (meth)acrylate – DMAEMA (Equation (1), mono(ethylene glycol) methacrylate – HEMA (Equation (2) and oligo(ethylene glycol) methacrylates (Equation (3) with various number of oxyethyl moieties (*n* = 6–22) and having terminal methoxy or hydroxyl groups.

In all three cases during hydrolysis there is a decrease in pH due to the formation of methacrylic acid (MAA) which affects the reaction rate. Therefore kinetics of the methacrylates hydrolysis is usually studied in the presence of buffer additives. Moreover, to eliminate the influence of additional factors (e.g., self-assembly of reagents) the studies are conducted in dilute solutions of esters. However, in practice the aqueous solutions of oligo(ethylene glycol)- and amine-containing methacrylates are used, as a rule, without buffer additives. Also, for technical and economic reasons, in practice, such monomers are typically obtained and used at high concentrations in water (10% or more). Therefore, we studied features of the hydrolysis of water-soluble methacrylic esters in a wide concentration range in the absence of buffer additives.

## 2. Experimental

### 2.1. Materials

The monomers DMAEMA and HEMA («Aldrich») were distilled twice in vacuum before use. Oligo(ethylene glycol) methacrylates («Cognis») of trademarks «Bisomer PEM 6 LD» (OEGMA-6, *n* = 6, R=H), «Bisomer MPEG 350 MA» (MOEGMA-8, *n* = 8, R=CH_3_), «Bisomer MPEG 550 MA» (MOEGMA-12, *n* = 12, R=CH_3_), «Bisomer S10 W» (MOEGMA-23, *n* = 23, R=CH_3_) were used without further purification. Distilled water was used as reaction medium in all experiments.

### 2.2. Experiment

**Figure 1.**
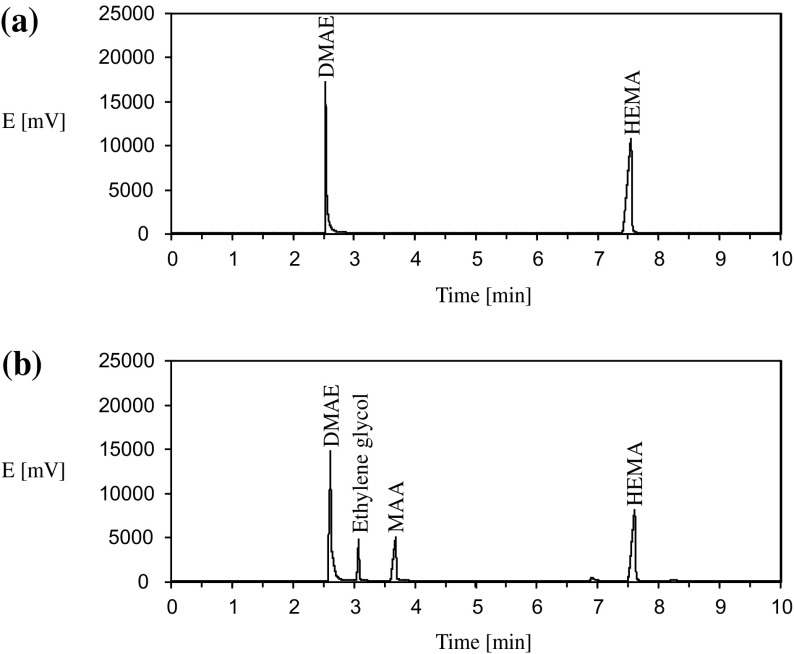
Examples of GC traces for the initial (a) and reaction (b) mixtures for hydrolysis of HEMA in the presence of DMAE (the initial concentrations of HEMA and DMAE – 0.4 mmol/g, *T* = 80 °C). (b) The reaction time – 40 min.

The hydrolysis of esters was studied in aqueous solutions in the temperature range of 20–80 °C. The initial monomer concentrations were varied from 0.2 to 5.0 mmol/g. To eliminate the radical polymerization of methacrylic monomers hydroquinone was added in the initial mixtures (0.3% by weight). Sodium hydroxide and 2-dimethylaminoethanol (DMAE) were used as external catalysts for hydrolysis. DMAE concentration in all experiments was constant (0.4 mmol/g), the concentration of NaOH was 0.2, 0.4 and 0.6 mmol/g. The degree of hydrolysis for all the monomers was determined using acid-base titration. Current concentrations of HEMA were determined by the gas chromatography (the examples of GC traces for the initial and reaction mixtures are shown in Figure 1); GC analysis was performed using a Chromos GH-1000 gas chromatograph with a flame-ionization detector and a capillary column VB-1701 (30 m × 0.32 mm × 0.5 um). The evaporator temperature was 240 °C; the chromatographic oven temperature varied according to the program 100 °C – 0 min – heating 5 °C/min to 220 °C; the detector temperature was 260 °C. Carrier gas – nitrogen, inlet pressure in the column 0.8 bar. The determination of OEGMAs concentrations was performed using a ‘Chromos LC-301’ liquid chromatograph with a UV-detector (column Perkin Elmer Amino Sil-X-1 0.46 × 25 cm, eluent – mixture of water, triethylamine (1 vol%) and phosphoric acid added to achieve pH 3). The kinematic viscosity of aqueous solutions of the monomers was measured using viscosimeter VPZh-2 at 25 °C.

## 3. Results and discussion

In the presence of sodium hydroxide the hydrolysis of the monomers occurs very rapidly even at room temperature (Table 1). Adding NaOH in dilute or concentrated solutions of HEMA after a few minutes leads to a rapid decrease in the concentration of the free alkali and the medium becomes neutral. This is due to the hydrolysis of HEMA and neutralization of the alkali by MAA formed. After binding of the entire free alkali the hydrolysis stops. Thus, the conversion of HEMA at the time of stopping the hydrolysis depends on the initial ratio of HEMA and alkali. In fact, the amount of HEMA undergoing the rapid hydrolysis is equivalent to the initial concentration of free alkali (see Table 1, Nos. 1–5).

**Table 1.** Effect of the initial concentrations of monomers (Cm, mmol/g) and the alkali (Ca, mmol/g) on the conversion of the esters in the reactions (1) and (2) in the presence of NaOH (*T* = 20 °C).

No.	Monomer	Cm	Ca	Time (min)^a^							
				3	5	10	20	30	40	50	70
1	HEMA	0.4	0.4	86.6 (86.6)	89.7 (89.7)	96.7 (96.7)	99.0 (99.0)	–	–	–	–
2	HEMA	2.5	0.4	15.8 (98.6)	–	15.9 (99.3)	15.9 (99.3)	15.9 (99.3)	–	–	–
3	HEMA	5.0	0.4	7.8 (97.6)	7.9 (98.8)	7.9 (98.8)	7.9 (98.8)	–	–	–	–
4	HEMA	0.4	0.2	43.3 (86.7)	–	49.5 (98.9)	49.5 (98.9)	49.9 (99.8)	49.9 (99.8)	–	–
5	HEMA	0.4	0.6	92.8 (61.9)	–	100 (66.7)	100 (66.7)	–	–	–	–
6	MOEGMA-12	0.4	0.4	89.0 (89.0)	90.1 (90.1)	94.9 (94.9)	96.9 (96.9)	97.9 (97.9)	98.4 (98.4)	98.6 (98.6)	–
7	MOEGMA-12	1.3	0.4	5.7 (18.4)	–	12.3 (39.9)	13.1 (42.8)	14.4 (46.7)	–	25.8(83.7)	30.3(98.4)
8	DMAEMA	0.4	0.4	68.3 (68.3)	79.9 (79.9)	88.0 (88.0)	91.6 (91.6)	94.5 (94.5)	94.7 (94.7)	95.5 (95.5)	96.8 (96.8)
9	DMAEMA	2.5	0.4	14.5 (90.8)	–	14.8 (92.4)	14.9 (93.1)	15.3 (95.4)	–	15.9 (99.4)	-
10	DMAEMA	5.0	0.4	0.5 (6.5)	–	–	1.5 (18.6)	2.9 (36.4)	4.3 (53.6)	5.3 (66.4)	7.0 (87.6)

^a^ The data on the conversion of the esters, %, and the degree of neutralization of the alkali, %, (in brackets) by methacrylic acid formed during hydrolysis with increasing reaction time.

A similar pattern is observed for the 50% aqueous solution of MOEGMA-12 (the mole monomer concentration of 0.4 mmol/g (Table 1, No. 6)): after 3 min the conversion of ester groups was 89%. However, when increasing the concentrations of oligo(ethylene glycol)- and amine-containing methacrylic esters the rate of their base-catalyzed hydrolysis is greatly reduced. This is shown by the example 7, in which the concentration of MOEGMA-12 was 1.3 mmol/g (or 82 wt.%). DMAEMA behaves similar: at the concentrations of 0.4 mmol/g (6.3 wt.%) and 2.5 mmol/g (39.3 wt.%) the conversion of about 90% is reached for 15 and 3 min, respectively, while at the concentration of 5.0 mmol/g (78.5 wt.%) the time to reach this level of the conversion is greater than 70 min.

These data are important in practical terms. They show that the addition of strong bases to the systems consisting of amine-containing or ethoxylated methacrylates can in a very short time lead to the complete consumption of these monomers with the formation of hydrolysis products even at room temperature; at elevated temperature the alkali-catalyzed hydrolysis should proceed more rapidly.

**Figure 2.**
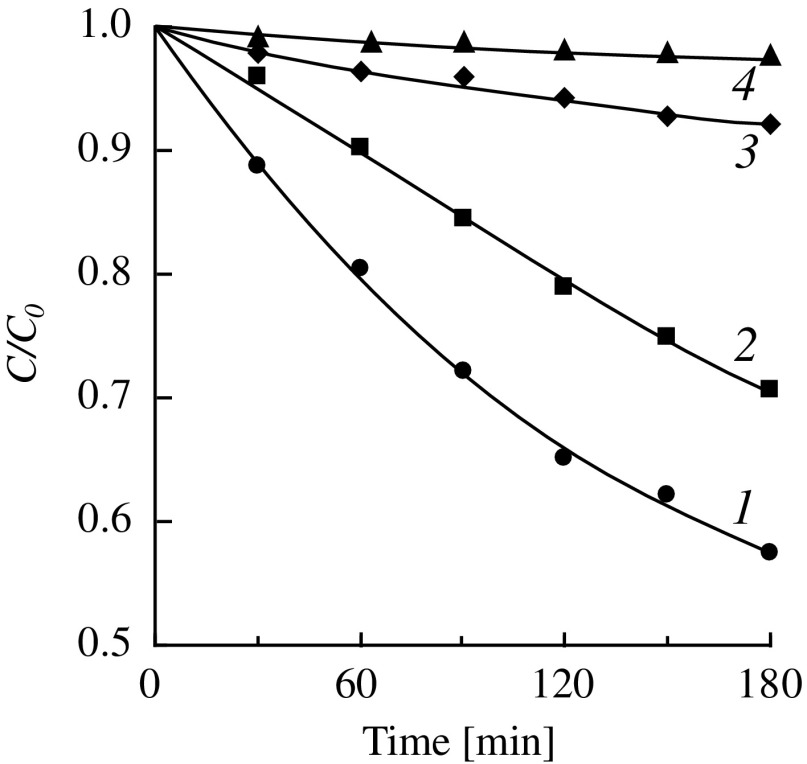
Kinetics of the autocatalytic hydrolysis of DMAEMA (25 °C). [DMAEMA]_0_, wt.% (mmol/g): 1 – 3.0 (0.191); 2 – 10.0 (0.637); 3 – 30.0 (1.911); 4 – 60.0 (3.822).

**Figure 3.**
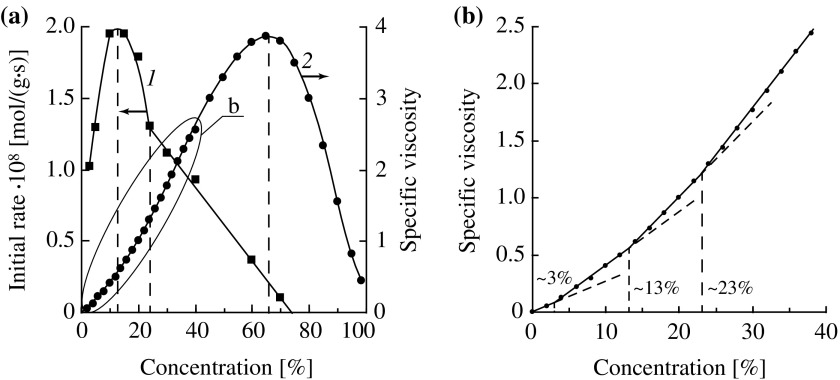
(a) Effect of the initial concentration of DMAEMA on the initial rate of hydrolysis (1) and the specific viscosity of aqueous solutions (2) (*T* = 25 °C); (b) the portion of the curve 2 in an enlarged scale.

When using amines for catalysis the reaction rate decreases sharply. At the same time for the monomers of different structure significant differences in the influence of the initial concentration on the rate of the reaction were observed. The autocatalytic hydrolysis of DMAEMA at 25 °C proceeds very slowly. At the concentration of DMAEMA of 10% the monomer conversion after 2 days was 90%, at the concentration of 70% after 10 days the conversion was 13%, and for 90% solutions of DMAEMA no hydrolysis was observed for 10 days. Kinetic curves of the low-temperature autocatalytic hydrolysis of DMAEMA are shown in Figure 2. Figure 3(a) shows the dependence of the initial rate of hydrolysis (IR_h_) on the initial monomer concentration ([*M*]_0_). This dependence has a maximum corresponding to the initial DMAEMA concentration of 12 wt.%, which corresponds to a mole ratio H_2_O:DMAEMA = 64:1. With further increase in the concentration a sharp decrease in the initial reaction rate is observed. This occurs despite the increase in the concentration of the catalytic amino groups and the fact that water is still in great excess with respect to DMAEMA. Therefore, the reasons for the sharp decrease in the rate of hydrolysis are not related to these factors.

We hypothesized that the structure of aqueous solutions of DMAEMA plays an important role in this case. It is known that DMAEMA molecules are capable of exhibiting surface activity [23] which indicates their propensity to self-assemble in water. Viscosity of solutions is one of the most sensitive parameters affected by changes in the structure of aqueous solutions. We obtained an experimental viscosity isotherm for aqueous solutions of DMAEMA. It has a pronounced maximum (Figure 3(a), curve 2) and several less pronounced inflection points delimiting line portions (Figure 3(b)). A similar kind of viscosity–concentration dependences of solutions of organic compounds is usually attributed to their propensity to self-assemble or aggregate with a solvent, and the presence of inflection points is explained by changes in the prevailing structure of aggregates.[24]

We found a clear correlation between a form of the viscosity isotherm for DMAEMA solutions and the concentration dependence of the hydrolysis rate. As it is seen from Figure 3(a), for dilute aqueous solutions of DMAEMA, with increasing the monomer concentration from 3 to 12–13% a close to linear increase in the initial hydrolysis rate occurs simultaneously with a linear increase in solution viscosity. The second inflection point on the viscosity isotherm corresponds to the maximum hydrolysis rate (Figure 3(b)). The third inflection point (the concentration of DMAEMA of about 23 wt.%) corresponds to an abrupt change in slope of the curve IR_h_ – [*M*]_0_ (Figure 3(a), curve 1). Finally, at the concentration of DMAEMA corresponding to the maximum viscosity the hydrolysis is almost completely stopped. Thus, these results show that the concentration dependence for the DMAEMA hydrolysis is connected with changes in the structure of aqueous solutions with increasing the monomer concentration. Such rearrangement of aqueous solutions of the investigated monomers was studied by us in detail in work.[20]

One can find analogies between the auto-catalyzed hydrolysis of DMAEMA and the known effect of the significant inhibition of the esters hydrolysis in dilute aqueous solutions in the presence of surfactants,[25,26] water-soluble polymers,[27] and hydrotropes.[28] Probably, in the absence of such external additives when increasing the concentration the molecules of water-soluble methacrylates gradually form structures that reduce the rate of hydrolysis. But only at high concentrations the structuring of the solution occurs which leads to the suppression of hydrolysis.

As mentioned above, in some cases, copolymers of ethoxylated methacrylates with aminoamide and aminoester monomers are obtained in aqueous solutions at elevated temperatures (typically around 80 °C). Therefore, we also studied the hydrolysis of HEMA and oligo(ethylene glycol) methacrylates catalyzed by an external water-soluble tertiary amine. As an amine we used DMAE forming during the hydrolysis of DMAEMA (Equation (1)). This tertiary amine is readily soluble in water and is a good model of industrial amine-containing (meth)acrylic monomers. It can not be hydrolyzed under experimental conditions because it contains no ester group, and its pKa value (9.3 [29]) is sufficiently close to that for water-soluble (meth)acrylic monomers containing tertiary amino groups.

**Figure 4.**
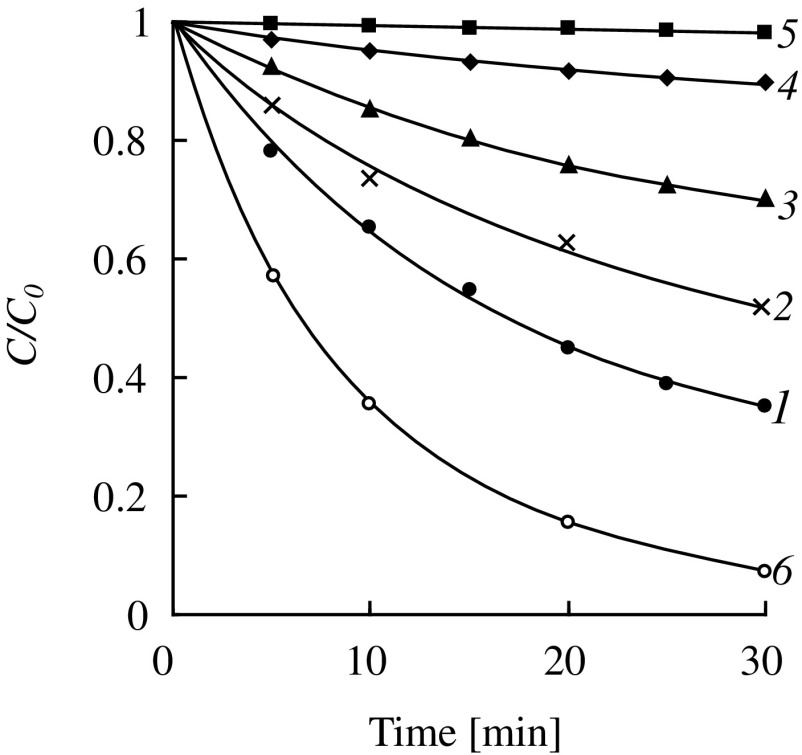
Kinetics of the hydrolysis of HEMA (1–5, catalyst – DMAE, 0.4 mmol/g) and DMAEMA (6, autocatalytic reaction) at the temperature of 80 °C. [HEMA]_0_, wt.% (mmol/g): 1 – 2.6 (0.20); 2 – 5.2 (0.40); 3 – 10.0 (0.77); 4 – 25.0 (1.92); 5 – 60.0 (4.62); [DMAEMA]_0_, wt.% (mmol/g): 6 – 6.3 (0.40).

As in the DMAEMA hydrolysis, the replacement of a strong base (NaOH) by a much weaker one (DMAE) significantly reduces the rate of the hydrolysis of OEGMAs. Examples of kinetic curves of the hydrolysis of HEMA at different initial monomer concentrations are shown in Figure 4. It is interesting to compare features of the hydrolysis of HEMA and DMAEMA under catalysis by the tertiary amino groups: the external and internal ones. HEMA can be considered as an analog of DMAEMA but containing hydroxyl group in the *β*-position of an alcohol part of the molecule instead of the tertiary amino group. It is known [30] that the negative inductive effect of an amino group is stronger than that of a hydroxyl group, therefore DMAEMA should have a carbonyl group with higher activity in the hydrolysis reaction. Indeed, in experiments with the same initial concentrations of the ester and amine groups, DMAEMA is hydrolyzed much faster than HEMA (Figure 4, curves 2 and 6). The dependences of the initial rate of hydrolysis on the monomer concentration for DMAEMA (Figure 3(a)) and HEMA (Figure 6, curve 1) are very similar – they have a maximum at the monomer concentration of about 12% while at the concentration of about 80% the rate of hydrolysis is close to zero. It should be noted, however, that in the presence of equal amounts of alkali DMAEMA is hydrolyzed more slowly than HEMA at any concentrations (especially in highly concentrated solutions) (see Table 1). Thus, the ratio of activities of these two monomers in the hydrolysis reaction catalyzed by alkali and amine vary. One possible reason for this can be the proximity of the amine catalytic center to the ester group in case of DMAEMA hydrolysis, which is absent in case of HEMA.

**Figure 5.**
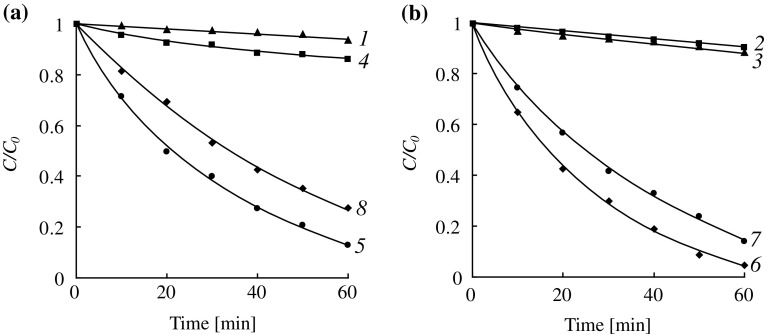
Effect of the initial concentration of oligo(ethylene glycol) methacrylates OEGMA-6 (1, 5), MOEGMA-8 (2, 6), MOEGMA-12 (3, 7), MOEGMA-22 (4, 8) on their consumption in the hydrolysis reaction (catalyst – DMAE, 80 °C). [Monomer]_0_, wt.%: 40.0 (1–4); 3.5 (5); 4.3 (6); 6.3 (7); 10.0 (8). The structures of (M)OEGMAs are presented in Scheme 1 and Section 2.1.

**Figure 6.**
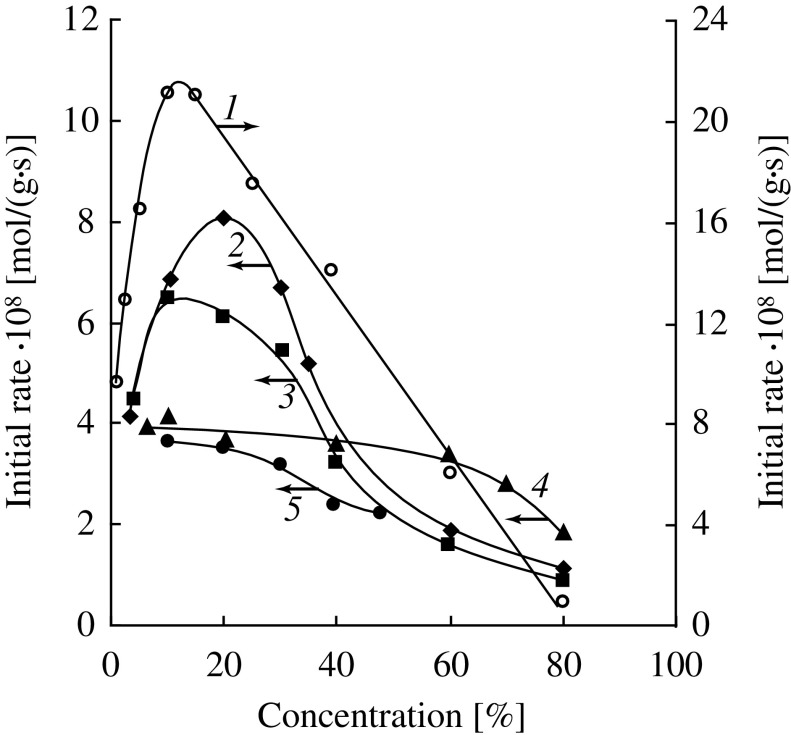
Dependence of the initial rate of the hydrolysis of HEMA (1), OEGMA-6 (2), MOEGMA-8 (3), MOEGMA-12 (4), MOEGMA-22 (5) on the initial concentration of the monomers (catalyst – DMAE, 0.4 mmol/g, 80 °C).

Further the concentration dependences for the hydrolysis of oligo(ethylene glycol) methacrylates were examined. Figure 5 shows kinetic curves of consumption of OEGMAs of different structure in the hydrolysis reaction catalyzed by DMAE (*T* = 80 °C). As examples the kinetic curves corresponding to different ranges of initial concentrations for each monomer were selected.

Summary data on the amine-catalyzed hydrolysis of (M)OEGMAs are shown in Figure 6. Several conclusions can be drawn. Firstly, when the monomer concentrations approach the minimum values the initial rates of the hydrolysis of different monomers tend to values of about 4 × 10^−8^ mol/(g s). This indicates close true reactivities of methacrylates regardless of the ethoxylation degrees and terminal group in the alcohol moiety. Secondly, when increasing the monomer concentrations the initial rates of the hydrolysis of various (M)OEGMAs begins to vary considerably. For the monomers HEMA, OEGMA-6, MOEGMA-8 the dependences IR_h_ – [*M*]_0_ have pronounced maxima corresponding to the monomer concentrations of 10–20 wt.%. For the monomers with higher degrees of ethoxylation (MOEGMA-12, MOEGMA-23) these dependences have no maxima. Thirdly, at the concentrations of higher than 20 wt.% the initial rate of hydrolysis begins to decrease sharply, and at the concentrations of about 80% it again becomes quite close for the monomers of different structure.

It is known that on going from aqueous solutions to aqueous-organic homogeneous solutions the strength of acids and bases can change considerably due to changes in the dielectric constant of medium and the structure of solutions. In particular, the ability of organic acids or bases to be involved in the formation of hydrogen bonds can be very important in this case.[31,32]

Molecules of (M)OEGMAs contain several oxygen atoms capable of participating in hydrogen bonding. We have shown a correlation between the types of hydrogen bonds formed by the ethoxylated methacrylates and the viscosity of solutions.[23] The reason for this correlation is the formation of highly structured solutions at high concentrations of (M)OEGMAs in which almost all water molecules are connected with monomer molecules by hydrogen bonds. This agrees completely with the results obtained in this work on the concentration effects in the hydrolysis of ethoxylated methacrylates. Hydrolysis is almost completely suppressed at high monomer concentrations, i.e., when forming structured solutions in which almost all of the monomer and water molecules are bound by hydrogen bonds. Oxygen atoms of carbonyl groups and oxyethyl moieties of HEMA and (M)OEGMAs participate in the formation of these bonds. The concentrations corresponding to the complete structuring of solutions and the maximum viscosity of solutions depend on the mole ratio of water to monomer and amount to 80–90 wt.% for the monomers of different structure. At such concentrations the rate of hydrolysis becomes negligible.

Binding of water molecules as a result of the structuring of solutions at high concentrations of (M)OEGMAs leads to a significant decrease in the actual concentrations of hydroxyl ions which catalyze hydrolysis. This was confirmed by other experiments in which investigated ethoxylated monomers were replaced by the compounds having ethoxylated fragments but having no hydrolyzable groups to eliminate the effect of the hydrolysis reaction. Ethyl cellosolve was chosen as a model compound for HEMA and polyethylene glycol of molecular weight 600 (PEG-600) and methoxypolyethylene glycol of molecular weight 750 (MPEG-750) for OEGMA-6 and MOEGMAs.

**Figure 7.**
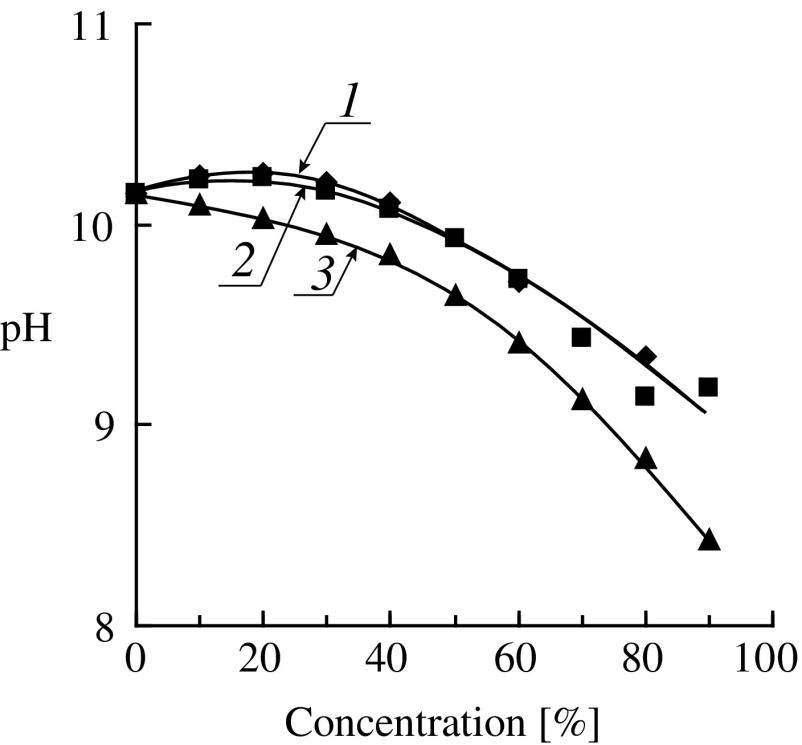
Dependence of pH of aqueous solutions on the concentration of PEG-600 (1), MPEG-750 (2) and ethyl cellosolve (3) at the constant concentration of DMAE (0.4 mmol/g), *T* = 80 °C.

Figure 7 shows the dependences of pH of aqueous solutions on the concentration of compounds that simulate molecules of (M)OEGMAs (at the constant concentration of the amine (DMAE)). These data confirm a significant decrease in the concentration of hydroxyl ions for the concentrated solutions of ethoxylated compounds.

**Scheme 1.**
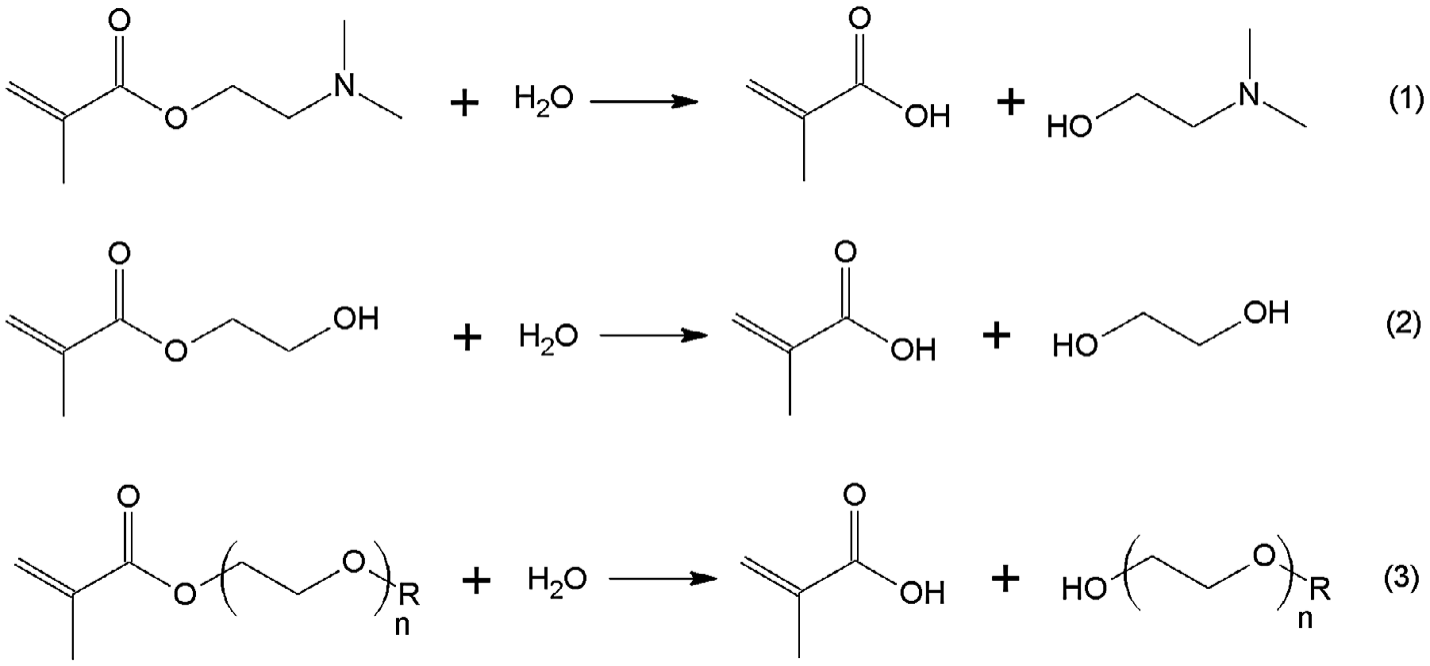
Hydrolysis reactions of DMAEMA (1), HEMA (2) and (M)OEGMAs (3).

Thus, the suppression of the hydrolysis of (M)OEGMAs is connected with a decrease in the mobility of reagents in the structured solutions as well as with a decrease in the actual concentrations of hydroxyl ions which catalyze hydrolysis.

## 4. Conclusions

In the presence of bases the water-soluble methacrylates (DMAEMA, HEMA, (M)OEGMAs) are rapidly hydrolyzed in aqueous solutions at room temperature, but the reaction rate decreases sharply in highly concentrated solutions of DMAEMA and (M)OEGMAs. Therefore, when carrying out industrial processes using water-soluble methacrylic esters one should take into account this factor as it can lead to changes in the actual composition of reaction mixtures and, as a consequence, to the deviation of the products composition from the expected one.

The suppression of the autocatalytic hydrolysis of DMAEMA in concentrated aqueous solutions is connected with the propensity of the monomer to self-assemble. For dilute solutions, methacrylic esters with the various number of oxyethyl moieties have almost no difference in activity in the hydrolysis reaction catalyzed by the water-soluble amine. At the monomer concentrations of 15–20% the hydrolysis rate significantly increases (for the monomers with low degrees of ethoxylation) or varies slightly (for highly ethoxylated monomers). At higher concentrations the hydrolysis rate is significantly reduced due to the structuring of solutions and decreasing the initial pH.

## Disclosure statement

No potential conflict of interest was reported by the authors.

## Funding

The work was supported by The Ministry of Education and Science of the Russian Federation (the study of hydrolysis in the presence of amines was funded by the project part of the state task in the field of scientific activity [grant number 10.1686.2014/K], the study of alkaline hydrolysis was funded by the contract No. [02.G25.31.0119]) and was performed using equipment of the Center for collective use ‘New materials and energy saving technologies’ of Lobachevsky State University of Nizhny Novgorod, project 14.594.21.0005 to MIL and EVS.
